# Proteomic Investigation in Plasma from Women with Fibromyalgia in Response to a 15-wk Resistance Exercise Intervention

**DOI:** 10.1249/MSS.0000000000002790

**Published:** 2021-09-22

**Authors:** KARIN WÅHLÉN, HONG YAN, CHARLOTTE WELINDER, MALIN ERNBERG, EVA KOSEK, KAISA MANNERKORPI, BJÖRN GERDLE, BIJAR GHAFOURI

**Affiliations:** 1 Pain and Rehabilitation Centre, and Department of Health, Medicine and Caring Sciences, Linköping University, Linköping, SWEDEN; 2 BioMS, Lund University, Lund, SWEDEN; 3 Department of Clinical Sciences Lund, Division of Oncology, Lund University, Lund, SWEDEN; 4 Department of Dental Medicine, Karolinska Institutet, and Scandinavian Centre for Orofacial Neurosciences (SCON), Huddinge, SWEDEN; 5 Department of Clinical Neuroscience, Karolinska Institutet, Stockholm, SWEDEN; 6 Department of Surgical Sciences, Uppsala University, Uppsala, SWEDEN; 7 Department of Neuroscience and Physiology, Section of Health and Rehabilitation, Physiotherapy, Sahlgrenska Academy, University of Gothenburg, Gothenburg, SWEDEN

**Keywords:** CHRONIC PAIN, IMMUNITY, MASS SPECTROMETRY, BIOMARKERS, BLOOD

## Abstract

**Purpose:**

Fibromyalgia (FM) is a complex pain condition, and exercise is considered the first option of treatment. Few studies have examined the effect of exercise on molecular mechanisms in FM. The aim of this study was to analyze the plasma proteome in women with FM and healthy controls (CON) before and after 15 wk of resistance exercise. This study further investigated whether clinical and exercises-related outcomes correlated with identified plasma proteins in FM.

**Methods:**

Plasma samples from 40 FM/25 CON (baseline) and 21 FM/24 CON (postexercise) were analyzed using shotgun proteomics. Clinical/background data were retrieved through questionnaires. Exercise-related variables and pressure pain thresholds were assessed using standardized instruments. Multivariate statistics were applied to analyze the proteomic profile at baseline and postexercise, and correlation with clinical/exercise-related data.

**Results:**

Fifteen weeks of resistance exercises improved clinical symptoms and muscle strength, and affected circulating proteins related to immunity, stress, mRNA stability, metabolic processes, and muscle structure development in FM. Pressure pain threshold was related to a specific protein profile, with proteins involved in metabolic and immune response. Subgroups of FM based on plasma proteins, FM duration, and improved muscle strength were identified.

**Conclusions:**

Exercise seems to affect circulating proteins, clinical characteristics, and muscle strength in FM. This study contributes to better understanding of systemic protein changes in FM compared with CON and how resistance exercise affects such changes.

## Abstract 2

Supplemental digital content is available in the text.

Fibromyalgia (FM) is a chronic musculoskeletal condition with a worldwide prevalence of 2.7% (1). FM symptoms include generalized musculoskeletal pain, stiffness, fatigue, anxiety, and depression, all conditions that have high impact on everyday life (2). The general treatment of FM is symptomatically based and includes both pharmacological and nonpharmacological options. The nonpharmacological strategies are often addressed in a multimodal setting including education, exercise interventions, workplace interventions, and psychological and behavioral therapies. Individuals with FM are reported to be less physically active (3), have reduced muscle strength (4,5), and experience fear-avoidance behavior toward exercise and potential worsening of symptoms (6,7). Nonetheless, physical exercise is considered one of the first options for treatment of FM and is in fact the only treatment that received a “strong for” recommendation among the European FM treatment recommendations (8). Different forms of exercise have shown positive effects on clinical symptoms such as pain intensity, muscle strength, fatigue, quality of life, and physical function in FM (9–12).

In healthy individuals, exercise improves muscle strength and quality of life, and helps to control body weight, blood pressure, blood glucose, insulin levels, and lipoprotein levels (13). Furthermore, exercise has a positive effect on the immune system as it alters immune cells such as leukocytes and releases cytokines, acute-phase proteins, and hormones (14). Exercise has been proposed to reduce systemic low-grade inflammation (15). A systemic review found that aerobic and resistance exercise was associated with either reduced or unchanged inflammatory markers (e.g., interleukin (IL)-6, IL-8, tumor necrosis factor α, C-reactive protein) in sedentary healthy subjects (16). Hence, studies on FM patients performing exercise interventions and evaluating different markers have been sparse. In studies investigating the cytokine profile of FM, modest alterations were found before and after aerobic exercise (17,18), strength/resistance exercise (19–21), aquatic exercise (22–24), and relaxation therapy/mindfulness (20,25). Furthermore, other metabolites such as glutamate and pyruvate were found to be normalized after 15 wk of resistance exercise in FM (26). In the same FM cohort, changes of plasma levels of endocannabinoids were found after resistance exercise (27). Finally, oxidative stress markers in FM have been shown to be reduced after 12 wk of combined aerobic and strength exercise (28). Overall, exercise is beneficial for FM regarding clinical symptoms and, to some extent, molecular markers; however, the mechanisms responsible for these effects are not fully elucidated.

Proteomics can be used to evaluate and explore potential known and unknown protein biomarkers in different biofluids or tissues. The analysis of systemic mechanisms, molecular pathways, and the proteome of plasma or serum from chronic pain patients and healthy controls can be used to investigate the effects of specific interventions. To date, proteomic studies evaluating the effect of exercise have mainly focused on male healthy subjects, for example, elite athletes subjected to extreme physical stress (29), young/elderly subjects performing aerobic exercise (30), and alterations in the serum proteome after high-intensity interval exercise (31). In these studies, the described protein alterations, seen systemically, are suggested to be involved in several processes, for example, of stress, immune response and inflammation, lipid clearance, and glucose–insulin transport.

The plasma/serum proteome has previously been investigated in chronic widespread pain (CWP) patients/FM and healthy controls (32–36), with the aim to explore protein changes related to the disease and relation to clinical manifestations of CWP/FM. However, to the best of our knowledge, no proteomic studies investigating the effect of resistance exercise on the plasma proteome in women with FM have been conducted.

The aim of this longitudinal study was to analyze the plasma proteome from FM patients before and after a 15-wk resistance exercise intervention. Furthermore, the aim was to investigate whether any of the clinical and exercise-related variables correlated with identified plasma proteins in FM. To better understand the changes of proteins after the exercise intervention, proteome differences between FM and CON at baseline (before the intervention) have also been investigated in this study.

## MATERIALS AND METHODS

### Overview of the Study

In the original study, women with FM and healthy controls (CON) were recruited to participate in a randomized multicenter study exploring the effects of 15 wk of resistance exercise intervention and relaxation therapy on women with FM (ClinicalTrials.gov NCTO1226784; October 21, 2010) (11,26). Details about the recruitment process have been well described in previous studies (11,37). After the initial recruitment process and completion of the baseline examination, 130 FM and 137 CON were included in the study. The FM group was randomized to either resistance exercise (*n* = 67) or relaxation therapy (*n* = 63), and the CON group was randomized to resistance exercise (*n* = 32) or baseline controls (*n* = 105; i.e., no intervention) (11,26).

For this study, plasma samples from 40 FM (23 resistance exercise and 17 relaxation therapy) and 25 CON (resistance exercise) were available for a cross-sectional proteomic analysis (before the start of the intervention). The longitudinal proteomic analysis, preexercise and postexercise, included participants only performing resistance exercise, with available plasma samples from both time points (the same participants were present in both preanalysis and postanalysis), resulting in 21 FM and 24 CON.

### Subjects

The study was conducted at three centers in Sweden (Gothenburg, Linköping, and Stockholm) and in accordance with the Declaration of Helsinki and Good Clinical Practice. The Ethical Review Board in Stockholm approved the study (Dnr: 2010/1121-31/3). The recruitment processes of all participants started in 2010, and data collection ended at all sites in 2013. Because of the timing of the study (initiated in 2009), the American College of Rheumatology (ACR) 1990 criteria were used (38).

The inclusion criteria for the CON group were women, age 20–65 yr, good health, and no current pain. The inclusion criteria for the FM group were women, age 20–65 yr, a confirmed FM diagnosis accessed by a physician using the ACR 1990 criteria, and available anamnesis. The exclusion criteria for both groups were high blood pressure (>160/90 mm Hg), osteoarthritis in the hip or knee, rheumatoid arthritis, systemic lupus erythematosus, psychiatric diseases (major depressive disorder, severe anxiety disorders, and psychosis), neurological diseases (e.g., multiple sclerosis and amyotrophic lateral sclerosis), diabetes mellitus, and cardiovascular diseases. In addition, the following exclusion criteria were used: primary causes of pain other than FM, high consumption of alcohol (audit >6), participation in a rehabilitation program within the past year, regular resistance exercise or relaxation therapy twice a week or more, inability to understand or speak Swedish, and not being able to refrain from analgesics (nonsteroidal anti-inflammatory drugs) or hypnotics for 48 h before examinations (11,37).

Before the start of the study, all participants received verbal and written information.

Written informed consent was obtained from all the participants. Each participant was compensated economically for their participation in the examinations.

### Intervention

The details for the resistance exercise program are described in a previous study (11). For both FM and CON groups, the resistance exercise intervention was focused on large muscle groups of the upper and lower extremities and the trunk as a way to improve core stability and power. Each training session was performed at the unit for physiotherapy at the respective pain clinic and was guided by an experienced physiotherapist, and the sessions included five to seven participants at a time (including only FM or CON). This approach was chosen to promote interactions between the participants and to facilitate guidance by the physiotherapist. Before the start of the intervention program, each participant had an individual meeting with the physiotherapist to discuss previous experiences and thoughts about exercise. In addition, assessment and adjustment of individual loads and confirmation of one-repetition maximum (1RM) for each assignment were performed. This information was used to design an individualized exercise program for each participant. For health and safety reasons, estimation of 1RM was assessed by letting the participant perform their maximum number of repetitions until exhaustion with an individually adjusted load. The intervention was initiated at 40% of 1RM, which was evaluated with the physiotherapist every third to fourth week to determine if loads needed to be increased. At the end of the intervention, each participant had reached between 60% and 80% of 1RM. The frequency of exercise was two times per week for 15 wk. The exercise program consisted of a 10-min warm-up (stationary bicycling) followed by a 50-min resistance exercise protocol. The resistance exercise program consisted of the following exercises: leg press, knee extension and knee flexion using weight machine, biceps curl and hand grip strength using free weights, heel raise and core stability using body weight, and 10 min of stretching. At 5 and 8 wk, explosive strength exercises were added: rapid heel raises and explosive knee extensions, respectively. The progression of the program was designed as follows: at baseline, 40% of 1RM for 15–20 repetitions for one to two sets; at 3 to 4 wk, 60% of 1 RM for 10–12 repetitions for one to two sets; and at 6 to 8 wk, 80% of 1RM for 5–8 repetitions for one to two sets. Between each set, there was a 1-min recovery period. If participants were unable to increase the loads to 80% of 1RM after 6 to 8 wk, they stayed at 60% of 1RM throughout the intervention (11).

### Background Data

Age (years), FM duration (years), systolic and diastolic blood pressure (mm Hg), and number of tender points were recorded. Weight and height were measured, and body mass index (BMI; kg·m^−2^) was calculated. These variables were only measured before the start of the intervention (i.e., at baseline).

### Clinical Variables

Each participant answered questionnaires regarding pain intensity, acceptance, fatigue, physical functioning and symptoms, catastrophizing, and psychological distress. Pressure pain thresholds (PPT) were assessed at baseline and postexercise. These instruments and measurements have been described in detail elsewhere, so hereinafter they are only briefly described (11,20).

Whole-body pain intensity for the past 7 d was reported using the visual analog scale (VAS global) ranging from 0 to 100 mm, where 0 is no pain and 100 is worst imaginary pain.

The Chronic Pain Acceptance Questionnaire (CPAQ-20), a measure of pain acceptance, consists of 20 items with a scale from zero to six for each item. The questionnaire is divided into two subscales that reflect activity engagement and willingness to experience pain. The sum of both subscales is reported as CPAQ total (0–120). A higher score indicates greater pain acceptance level.

The Multidimensional Fatigue Inventory (MFI) instrument was used to measure different dimensions of fatigue. MFI consists of 20 items that measures the following five dimensions: motivation, activity, general fatigue, physical fatigue, and mental fatigue. Each subscale is scored from 4 to 20; a higher score indicates more fatigue.

The Fibromyalgia Impact Questionnaire (FIQ) measures health status and consists of 10 items divided into subscales reflecting physical functioning and symptoms. Each item has a score from 0 to 100, and the mean sum including all 10 subscales is presented as the FIQ total score. A higher score indicates greater impairment.

The Pain Catastrophizing Scale (PCS) consists of 13 items divided into three subscales that measure rumination, helplessness, and magnification. The total score of the three subscales, which range from 0 to 52, is presented. A higher score implies worse catastrophizing thoughts.

The Hospital Anxiety and Depression Scale (HADS) consists of 14 items divided into two subscales that measure anxiety and depression. Each subscale contains seven items, with a possible score of 0–21. In this study, the total score of each subscale is summed and reported as HADS total with a range of 0–42. A higher score indicates a larger psychological distress.

The PPT was assessed using a pressure algometer (Somedic Sales AB, Höör, Sweden) as described in detail in previous studies (20,21). Briefly, the PPT was assessed across eight sites on both the right and left sides that represent tender points as defined by the ACR 1990 criteria: the supraspinatus muscle (at origins above the scapula spine near the medial border), the lateral epicondyle (2 cm distal to the epicondyles), the gluteus maximus (in the upper outer quadrants of the buttocks in the anterior fold of muscle), and the inside of the knee (at the medial fat pad proximal to the joint line). The mean value of all sites is presented as PPT mean.

### Measurement of Physical Capacity

The following parameters were measured to assess physical capacity at baseline and postexercise. All parameters and measurements are described in detail elsewhere (11,20).

The 6-min walk test (SixMWT) reflects physical performance and measures the total walking distance over 6 min in a standardized setting. The isometric force in the quadriceps muscles (knee extension force (N)) was measured using Steve Strong® dynamometer (Stig Starke HB, Gothenburg, Sweden). The maximum force was tested for 5 s, and the best result of three trials was recorded. The handgrip force was assessed using Grippit® dynamometer (AB Detektor, Gothenburg, Sweden). The test was performed over 10 s during two trials for each hand. The mean force (N) from both trials was recorded. The isometric strength in the elbow was measured using Isobex® dynamometer (Medical Device Solutions AG, Oberburg, Switzerland). The maximum strength (kg) was tested for 5 s, and the best result of three trials was recorded (11,20).

### Blood Collection

For both FM and CON, venous blood samples were collected using a Vacutainer (BD Vacutainer Eclipse Blood Collection Needle; BD Diagnostics, Becton, Dickinson, and Company, Franklin Lake, NJ) in 10 mL K_2_EDTA tubes (BD Vacutainer Plus Plastic K_2_EDTA Tubes, BD Diagnostics). The plasma was retrieved by centrifuging the blood samples for 30 min at 1500*g* at RT immediately after collection. The plasma fraction was removed to a new tube before aliquoted and stored in −86°C until analysis. The retrieval of the blood samples at baseline was performed after the clinical examination, within 1–7 d before the start of the intervention and within 1–7 d after the intervention.

### Proteomics

#### Sample preparation

All samples were blinded before the start of sample preparation. To check the sample preparation and instrument performance, individual plasma samples from each participant (40 FM/25 CON baseline, 21 FM/24 CON post) and a quality control sample including a pool of all included plasma samples from each participant were prepared and run together. All sample preparation steps, including desalting and protein digestion, used the Agilent AssayMAP Bravo Platform (Agilent Technologies, Inc.) per the manufacture’s protocol. Each plasma sample was diluted 1:10 (100 mM ammonium bicarbonate (AmBic); Sigma-Aldrich Co., St. Louis, MO), and 10 μL of each diluted plasma sample was transferred to a 96-well plate (Greiner G650201), where 40 μL of 4 M urea (Sigma-Aldrich) in 100 mM AmBic was manually added with a pipette for a final volume of 50 μL. The proteins were reduced with 10 μL of 60 mM dithiothreitol (final concentration of 10 mM; Sigma-Aldrich) for 1 h at 37°C followed by alkylation with 20 μL of 80 mM iodoacetamide (final concentration of 20 mM; Sigma-Aldrich) for 30 min in a dark at room temperature. The plasma samples were digested with Lys-C (FUJIFILM Wako Chemicals USA Corporation) with an enzyme to protein ratio of 1:50 wt/wt for 5 h at room temperature and further digested with trypsin (Sequencing Grade Modified; Promega, Madison, WI) with a trypsin-to-protein ratio of 1:50 wt/wt overnight at room temperature (39). The digestion was stopped by pipetting 20 μL of 10% trifluoroacetic acid (TFA; Sigma-Aldrich), and the digested peptides were desalted on Bravo platform. To prime and equilibrate the AssayMAP C18 cartridges (Agilent, PN: 5190-6532), 90% acetonitrile (ACN; Sigma-Aldrich) with 0.1% TFA and 0.1% TFA were used, respectively. The samples were loaded into the cartridges at the flow rate of 5 μL·min^−1^. The cartridges were washed with 0.1% TFA before the peptides were eluted with 80% ACN/0.1% TFA. The eluted peptides were dried in a SpeedVac (Concentrator plus Eppendorf) and resuspended in 25 μL of 2% ACN/0.1% TFA. The peptide concentration was measured using the Pierce Quantitative Colorimetric Peptide Assay (Thermo Fisher Scientific, Rockford, IL). The samples were diluted to 0.5 μg·μL^−1^ with 2% ACN/0.1% TFA and spiked with synthetic iRT peptides (JPT Peptide Technologies, GmbH, Berlin, Germany) before liquid chromatography–mass spectrometry (LC-MS/MS) analysis. One microgram of peptides was injected into the LC-MS/MS for the sample analysis. Quality control samples were analyzed after every 12th sample.

#### Samples used to build library

Ten randomly picked-up FM baseline and FM post samples were pooled. The quality control sample, the pooled FM baseline, and the pooled FM post sample were depleted and then fractionated to reduce the dynamic range and increase the coverage of the plasma proteome. The pooled samples were depleted with High Select™ Top14 Abundant Protein Depletion Mini Spin Columns (Thermo Fisher Scientific) according to the instructions supplied by the manufacturer, followed by the buffer exchange to urea and protein reduction with 10 mM dithiothreitol and alkylation with 20 mM iodoacetamide on an Amicon Ultra 0.5 mL 10 kDa cutoff spin column (Merck & Co., Inc., d.b.a.). The proteins were then transferred to new tubes and digested with trypsin overnight with an enzyme-to-protein ratio of 1:50. Protein digestion was quenched with 10% TFA. Two microliters of iRT peptides was spiked into 20 μL of each sample before LC-MS/MS analysis.

The depleted plasma digests were further fractionated with Pierce™ High pH Reversed-Phase Peptide Fractionation Kit (Thermo Fisher Scientific) according to the manual supplied by the manufacturer, and the peptides were eluted in a stepwise fashion with 5%, 7.5%, 10%, 12.5%, 15%, 17.5%, 20%, and 50% ACN in 0.1% triethylamine. All the fractions were dried by SpeedVac concentrator. The samples were resuspended in 20 μL of 2% ACN/0.1% TFA and spiked with 2 μL of iRT peptides before LC-MS/MS analysis.

#### LC-MS/MS analysis

The plasma samples were analyzed on Hybrid mass spectrometer Q Exactive HF-X (Thermo Fischer Scientific) coupled with an Ultimate 3000 UHPLC system (Thermo Fischer Scientific). Two-column setup was used on the HPLC system, and peptides were loaded into an Acclaim PepMap 100 C18 precolumn (75 μm × 2 cm; Thermo Scientific, Waltham, MA) and then separated on an EASY spray column (75 μm × 50 cm, nanoViper, C18, 2 μm, 100 Å) with the flow rate of 300 nL·min^−1^. The column temperature was set to 60°C. Solvent A (0.1% FA in water) and solvent B (0.1% FA in 80% ACN) were used to create a gradient, and a 90-min linear gradient from 4% to 38% of solvent B in solvent A was used to elute the peptides.

#### Data-independent acquisition analysis

The Q Exactive HF-X was operated in the data-independent acquisition (DIA) mode, and the instrument method was adopted from previous published work (40) and optimized for 90-min gradient analysis. Full MS survey scans from *m*/*z* 350–1205 with a resolution 120,000 were performed in the Orbitrap detector. The automatic gain control (AGC) target was set to 3 × 10^6^ with the maximum injection time of 100 ms. One segment for MS1 was kept constant. For MS2, 26 segments with variable isolation windows were acquired with the resolution of 45,000. The normalized collision energy for higher energy collision dissociation and the AGC target for MS2 were 27 and 3 × 10^6^, respectively. The maximum injection time was set auto.

#### DDA analysis for the depleted samples and depleted and fractionated samples to build library

One nondepleted plasma sample, six depleted samples (quality control sample, FM baseline, FM post in duplicates), and 8 fractions of three depleted samples (in a total of 24 fractions from one quality control sample, one FM baseline and one FM post) were analyzed on Q Exactive HF-X with the positive data-dependent acquisition (DDA) mode. The full MS resolution was set to 120,000 at *m*/*z* 200, and the AGC target was 3 × 10^6^ with the maximum injection time of 100 ms. The full mass range was set at 375–1500 *m*/*z*. Precursors were isolated with the isolation window of 1.2 *m*/*z* and fragmented by higher energy collision dissociation with the normalized collision energy of 28. MS2 was detected in the Orbitrap with the resolution of 15,000. The AGC target and the maximum injection time were set at 1.0 × 10^5^ and 50 ms, respectively.

#### Spectral library generation with Spectronaut

All the acquired DDA and DIA raw data were loaded into Spectronaut Pulsar X (version 13; Biognosys, Schlieren, Switzerland) for the spectral library generation. The Uniprot Human FASTA file (downloaded on 20191203; entries: 42410), including isoforms and cysteine mutations, was used as protein database, and the default settings of Spectronaut library generation functionality were used for database match including full trypsin specific cleavage and a peptide length of between 7 and 52 amino acids. A maximum of missed cleavage was two, and a maximum of five variable modifications per peptide were allowed. Carbamidomethylation of cysteine residues was set as fixed modification, and acetyl (Protein-N-term), oxidation (M), oxidation (P), and phospho (STY) were set as variable modifications. All false discovery rates (FDRs) for the peptide-spectrum match, peptide, and protein were set at 0.01. The library was filtered before data analysis. Fragment ions with at least three amino acids (mass range from 300 to 1800 *m*/*z*) and with a minimum relative intensity of 5% were retained in the library. Fragment ions were selected based on the intensity, and the 3–6 most intensive fragments per peptide were included in the library. The iRT calibration was required with a minimum *R*^2^ of 0.8.

DIA data were analyzed comparing Spectronaut data with the built spectral library. The data were extracted based on maximum intensity for both precursors and fragment ions. The following default settings were applied for peptide and protein identification and quantification: excluding duplicate assays, generation decoy based on mutated method at 10% of library size, and estimation of FDRs using *Q* value as 0.01 for both precursors and proteins. The *P* value was calculated using a kernel-density estimator. For the quantification, interference correction was activated, and a minimum of three fragment ions and two precursor ions were kept. The area of MS2 level was used for quantitation. Peptide (stripped sequence) quantity was calculated by the mean of the 1–3 most intensive precursors, and protein quantity was calculated by the mean of 1–3 best peptides. Data were filtered by *Q* value, and cross-run normalization was inactive. The data were exported from Spectronaut, and downstream statistical analysis was performed.

### Statistics

#### Background, clinical, and exercise-related data

All data were tested for normality using the Shapiro–Wilk test, and thereafter either Mann–Whitney *U* or Student’s *t*-test was conducted for between-group comparison (FM vs CON). For within-group comparison (baseline vs postexercise), Wilcoxon signed rank test or paired-sample *t*-test was used. All statistical calculations were performed in SPSS version 25 (International Business Machines Corporation (IBM), Armonk, NY).

A *P* value ≤0.05 was considered significant. Data are presented as median and interquartile range (IQR) if not stated otherwise.

#### Multivariate statistics

The details for multivariate data analysis (MVDA) including principal component analysis (PCA) and quality checking data for outliers, orthogonal partial least square-discriminant analysis (OPLS-DA), and OPLS modeling using SIMCA version 15 (Sartorius Stedim Data Analytics AB, Umeå, Sweden) are described in detail elsewhere (34,41). Briefly, data were scaled with unit variance scaling, mean centered, and log transformed. Each OPLS-DA and OPLS model were performed in two steps as described previously (34,41). First, an initial model was created, including all identified and quantified proteins as *x*-variables. Next, the proteins with a variable influence on projection (VIP) value >1.0 were selected and used in a second regression model, which is presented in the Results section. The following parameters are presented for each MVDA model: number of principal (PC) and/or orthogonal components (OC), VIP or VIPpredictive (VIPpred), and p(corr). Goodness of fit and goodness of prediction for the model were expressed as *R*^2^ and *Q*^2^, respectively. To validate the significant level of each OPLS/OPLS-DA model, analysis of variance of cross-validated predictive residuals (CV-ANOVA) was used; *P* ≤ 0.05 was regarded as a significant model. In addition, a protein with a VIP or VIPpred value ≥1.0 and an absolute p(corr) of ≥0.35 was regarded as significant in each MVDA model. Stated p(corr) values refer to absolute p(corr) in the following text. For a detected protein to be included in the MVDA analysis, it had to be present in ≥40% in any of the groups.

#### Protein pathway analysis

The online database tool STRING version 11 (https://string-db.org/) was used to create and investigate functional enriched protein–protein association networks (42). The protein accession number (UniProt) from respective significant protein from each MVDA model was entered into the search engine of multiple proteins, and the following parameters were chosen: organism *Homo sapiens*, the maximum number of interactions was query proteins only, interaction score was set to high confidence (0.700), and an FDR of ≤0.05 was used when classifying the Gene Ontology biological process of each protein in the functional enriched network. When several known isoforms of a protein are present, the main accession number was used.

#### R analysis

To illustrate relative protein differences, a radar plot was created using R version 4.1.0 (43)/R Studio version 1.4.1717 (44) and the package *fmsb* version 0.7.1 (45).

## RESULTS

### Background, Clinical, and Exercise-Related Data

#### Baseline comparison FM versus CON

No significant differences were found in the background variables age or blood pressure between FM and CON, although FM had a significant higher BMI compared with CON (Table 1). Compared with CON, FM had significantly higher pain intensity (VAS score), fatigue (MFI all subscales), FIQ total score, PCS score, and HADS total score and significantly lower CPAQ score and PPT (Table 1). Significantly reduced physical capacity was found in FM compared with CON for the variables SixMWT, knee extension force right side, handgrip force (both sides), and elbow strength (both sides; Table 1). The FM and CON participants who performed resistance exercise (21 for FM and 24 for CON) had similar BMI (median (IQR), 25.3 (5.5) for FM and 24.3 (5.6) for CON; *P* = 0.172).

**TABLE 1:** Background, pain characteristics, and exercise-related data at baseline for FM and CON.

Variables	FM/CON	FM	CON	*P*
	(*N* Participants Included)	Median (IQR)	Median (IQR)	
Background data				
Age (yr)	40/25	52.0 (12.8)	52.0 (21.0)	0.513*^a^*
BMI (kg·m^−2^)	40/25	25.7 (4.9)	24.3 (5.5)	**0.049** ***^a^***
FM duration (yr)	40	10.5 (10.0)	—	—
Tender points (*n*)	40	16.0 (2.8)	—	—
Systolic blood pressure (mm Hg)	39/25	120.0 (30.0)	125.0 (20.5)	0.805*^b^*
Diastolic blood pressure (mm Hg)	39/25	85.0 (15.0)	85.0 (12.0)	0.726*^b^*
Pain characteristics variables				
VAS global pain (0–100)	40/25	54.5 (39.5)	0.0 (0.0)	**<0.00**1**^*a*^**
CPAQ total (0–120)	40/12	64.5 (22.8)	85.5 (42.5)	**<0.01** ***^b^***
MFI (4–20)				
General fatigue	40/25	19.0 (3.0)	8.0 (5.0)	**<0.001** ***^a^***
Physical fatigue	40/25	16.5 (3.8)	7.0 (4.0)	**<0.001** ***^a^***
Mental fatigue	40/25	16.0 (3.8)	6.0 (5.0)	**<0.001** ***^a^***
Reduced activity	40/25	14.5 (6.0)	5.0 (3.5)	**<0.001** ***^a^***
Reduced motivation	40/25	10.0 (6.5)	5.0 (2.0)	**<0.001** ***^a^***
FIQ total (0–100)	40/22	58.3 (22.5)	3.6 (8.9)	**<0.001** ***^a^***
PCS total (0–52)	40/17	18.0 (15.8)	3.0 (10.5)	**<0.001** ***^a^***
HADS total (0–42)	40/25	15.0 (13.8)	4.0 (5.0)	**<0.001** ***^a^***
PPT mean all sites (kPa)	40/25	153.5 (87.5)	357.0 (168.0)	**<0.001** ***^a^***
Exercise-related variables				
SixMWT (m)	40/24	567.0 (105.5)	647.0 (98.5)	**<0.001** ***^b^***
Steve Strong, knee extension force, right (N)	40/24	299.0 (128.0)	399.5 (90.3)	**<0.01** ***^b^***
Steve Strong, knee extension force, left (N)	40/24	299.0 (130.5)	369.5 (124.3)	0.058*^b^*
Grippit, handgrip force mean, right (N)	40/24	154.0 (101.5)	234.0 (41.5)	**<0.001** ***^b^***
Grippit, handgrip force mean, left (N)	40/24	152.5 (104.8)	210.0 (70.3)	**<0.001** ***^b^***
Isobex, elbow strength maximum, right (kg)	40/16	11.8 (7.9)	18.4 (7.8)	**<0.001** ***^b^***
Isobex, elbow strength maximum, left (kg)	40/16	11.9 (8.3)	17.3 (6.6)	**<0.001** ***^b^***

Presented data include all FM and CON participants at baseline. These data have been published elsewhere, although not the same number of participants (11,20). Bold font indicates significance (*P* < 0.05).

*^a^*Mann-Whitney *U* test.

*^b^*Student’s *t*-test.

#### Postexercise comparison between and within groups

Postexercise, the FM group significantly improved their general and mental fatigue, PCS score, and HADS total score. In addition, this group exhibited significant higher CPAQ score and reduction in pain intensity (Table 2). No significant improvement in PPT was seen. The FM group significantly improved their physical capacity in the variables knee extension force left side, handgrip force (both sides), and elbow strength right side (Table 2). In comparison with CON, the significant differences between the two groups remained in several of the clinical variables after exercise; for example, higher pain intensity, fatigue, psychological distress, pain catastrophizing, and pain sensitivity were seen in the FM group. Furthermore, physical capacity and hand strength remained reduced in the FM group compared with CON. The CON group improved their physical fatigue, walking distance, and knee and handgrip force (left side) after exercise. For more details, see Table S1 in Appendix (Supplemental Digital Content, http://links.lww.com/MSS/C433).

**TABLE 2:** Changes in pain characteristics and exercise-related variables in FM after exercise.

Variables	No. FM Included in Test Baseline and Postexercise	FM, Median (IQR)Baseline	FM, Median (IQR)Post	*P*
Pain characteristics variables				
VAS global pain (0–100)	21/21	54.0 (36.0)	35.0 (36.5)	**0.031** ***^a^***
CPAQ total (0–120)	21/21	62.0 (25.5)	75.0 (25.0)	**0.011** ***^b^***
MFI (4–20)				
General fatigue	21/21	19.0 (3.0)	18.0 (4.5)	**0.046** ***^a^***
Physical fatigue	21/21	16.0 (3.5)	14.0 (6.0)	0.095*^a^*
Mental fatigue	21/21	17.0 (3.5)	15.0 (7.0)	**0.024** ***^a^***
Reduced activity	21/21	16.0 (6.5)	15.0 (6.5)	0.120*^a^*
Reduced motivation	21/21	12.0 (6.0)	10.0 (8.0)	0.246*^a^*
FIQ total (0–100)	21/21	64.1 (22.1)	60.1 (29.2)	0.079*^a^*
PCS total (0–52)	21/21	20.0 (17.5)	13.0 (11.5)	**<0.01** ***^a^***
HADS total (0–42)	21/21	17.0 (14.0)	11.0 (17.5)	**0.047** ***^a^***
PPT mean all sites (kPa)	21/21	146.0 (116.0)	159.0 (105.0)	0.498*^a^*
Exercise-related variables				
SixMWT (m)	21/21	588.0 (127.5)	594.0 (122.5)	0.923*^b^*
Steve Strong, knee extension force, right (N)	21/21	330.0 (144.5)	410.0 (166.0)	0.065*^b^*
Steve Strong, knee extension force, left (N)	21/21	312.0 (175.0)	357.0 (152.0)	**0.044** ***^b^***
Grippit, handgrip force mean, right (N)	21/20	153.0 (137.5)	198.0 (116.8)	**<0.01** ***^b^***
Grippit, handgrip force mean, left (N)	21/20	150.0 (120.0)	169.5 (93.5)	**0.024** ***^b^***
Isobex, elbow strength maximum, right (kg)	21/21	11.9 (7.3)	14.7 (5.4)	**0.014** ***^b^***
Isobex, elbow strength maximum, left (kg)	21/21	11.5 (8.6)	14.5 (5.9)	0.113*^b^*

Presented data include FM participants who performed 15-wk resistance exercise intervention (*n* = 21) and which plasma samples were analyzed with proteomics at both time points. These data have been published elsewhere although not the same number of participants (11,20). Bold font indicates significance (*P* < 0.05).

*^a^* Wilcoxon signed rank test.

*^b^* Paired *t*-test.

#### MVDA analysis of LC-MS/MS data

In total, 575 proteins were identified and quantified in the plasma samples from the present cohort. Of the 575 proteins, 91 proteins were excluded because of missing values in ≥60% in respective group (i.e., FM and CON at baseline and postexercise), which resulted in inclusion of 484 proteins in the MVDA analysis. An initial PCA including all proteomic data and all observations was created to check for potential outliers; three CON and two FM observations were flagged as suspected outliers. After critically reviewing the data, no support for exclusion was found, and therefore, the observations were kept for future analysis (data not shown).

Several of the identified proteins are expressed as different known isoforms and contain identical peptides, which cannot be differentiated by the MS/MS data alone. Therefore, they were grouped. In each table, the proteins of concern are marked in bold. For full protein names and potential isoforms, see Table S2 (SDC, Appendix, http://links.lww.com/MSS/C433).

#### Pain-related proteomics (baseline comparison, FM vs CON)

**FIGURE 1:**
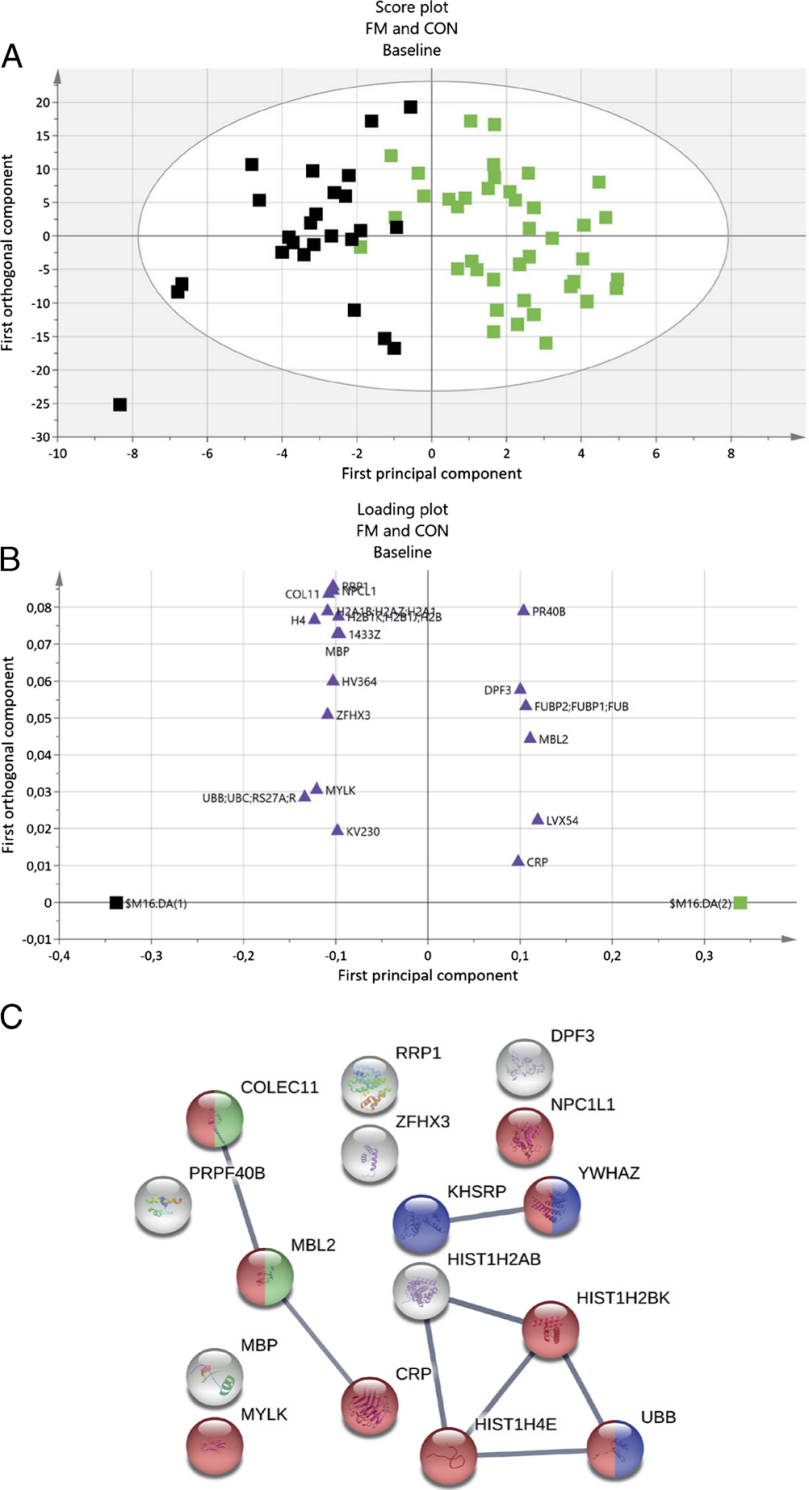
OPLS-DA model of FM and CON at baseline, and STRING protein network. A, Score plot visualizing group separation between FM and CON: green, FM; black, CON. B, Loading plot showing significant proteins with a VIPpred ≥1.0 and a p(corr) ≥0.35 (purple triangles). In total, 19 proteins were significantly important for group separation. Six of 19 proteins had a positively association with FM. C, Enriched STRING protein network of significant proteins. Node colors represent the following biological process: red, response to stress; green, complement activation, lectin pathway; blue, regulation of mRNA stability. Abbreviated protein names in loading plot and STRING network correspond to the same names in Table 3 and Table S2 (Supplemental Digital Content, Appendix, http://links.lww.com/MSS/C433).

A comparative proteomic analysis where the plasma proteome between FM and CON at baseline was investigated using OPLS-DA. The OPLS-DA model had one PC and two OC, *R*^2^ = 0.686, *Q*^2^ = 0.30, and CV-ANOVA *P* = 0.0028. This significant model had 19 proteins with a VIPpred ≥1.0 and a p(corr) ≥0.35. These proteins were regarded as significantly altered proteins important for group separation between FM and CON (Table 3). The proteins with highest VIPpred (≥1.66) were upregulated proteins of mannose-binding protein C and immunoglobulin lambda variable 10–54 and downregulated proteins of polyubiquitin-B, histone H4 (H4), myosin light-chain kinase (smooth muscle), zinc finger homeobox protein 3 (ZFHX3), and histone H2A type 1-B/E (H2A1B; Table 3). Of the 19 proteins, 6 were positively associated with FM and 13 were positively associated with CON (Figs. 1A, B).

**TABLE 3:** Significant proteins group differences between FM and CON at baseline.

Protein Name	Accession Number (Uniprot)	Protein Name in MVDA Model	Gene Name (STRING Analysis)	VIPpred	p (corr)	No. FM/CON Protein Is Detected	FM, Median (IQR) Intensity	CON, Median (IQR) Intensity	Fold Change FM/CON
**Polyubiquitin-B**	**P0CG47**	UBB	UBB	2.03	−0.67	12/11	3142 (2455)	7842 (7241)	0.40
Histone H4	P62805	H4	HIST1H4E	1.86	−0.44	40/25	65,140 (42,406)	65,789 (9478)	0.99
**Myosin light-chain kinase**, **smooth muscle**	**Q15746**	MYLK	MYLK	1.83	−0.44	40/25	3,387,278 (4,271,559)	5,315,174 (2,820,108)	0.64
*Immunoglobulin lambda variable 10–54	A0A075B6I4	LVX54	—	1.81	0.35	33/21	8923 (8906)	6654 (7154)	1.34
*Mannose-binding protein C	P11226	MBL2	MBL2	1.68	0.40	40/25	34,118 (34,718)	24,066 (32,527)	1.42
Zinc finger homeobox protein	Q15911	ZFHX3	ZFHX3	1.66	−0.39	40/25	520,290 (181,228)	602,514 (96,569)	0.86
**Histone H2A type 1-B/E**	**P04908**	H2A1B	HIST1H2AB	1.66	−0.38	37/25	21,929 (17,326)	26,473 (15,365)	0.83
**Collectin-11**	**Q9BWP8**	COL11	COLEC11	1.64	−0.39	40/25	10,188 (5180)	13,337 (6129)	0.76
*Far upstream element-binding protein 2	**Q92945**	FUBP2	KHSRP	1.60	0.37	40/25	49,207 (24,240)	38,046 (25,907)	1.29
*Pre-mRNA-processing factor 40 homolog B	**Q6NWY9**	PR40B	PRPF40B	1.58	0.38	39/25	10,152 (8760)	9258 (9339)	1.10
Immunoglobulin heavy variable 3–64	A0A075B6Q5	HV364	—	1.57	−0.37	37/22	41,827 (71,259)	79,534 (72,831)	0.53
Ribosomal RNA processing protein 1 homolog A	P56182	RRP1	RRP1	1.57	−0.37	40/25	51,370 (17,162)	55,964 (19,092)	0.92
**NPC1-like intracellular cholesterol transporter 1**	**Q9UHC9**	NPCL1	NPC1L1	1.56	−0.37	40/25	246,211 (89,713)	249,761 (73,327)	0.99
*Zinc finger protein DPF3	**Q92784**	DPF3	DPF3	1.52	0.35	40/25	8607 (3946)	8381 (4285)	1.03
Immunoglobulin kappa variable 2–30	P06310	KV230	—	1.50	−0.35	40/25	16,695 (9777)	21,931 (11,686)	0.76
*C-reactive protein	P02741	CRP	CRP	1.49	0.37	33/21	16,860 (41,509)	9780 (19,697)	1.72
**Myelin basic protein**	**P02686**	MBP	MBP	1.49	−0.35	40/25	12,014 (6873)	11,432 (1619)	1.05
**Histone H2B type 1-K**	**O60814**	H2B1K	HIST1H2BK	1.48	−0.35	40/25	28,789 (14,691)	29,352 (9473)	0.98
14-3-3 protein zeta/delta	P63104	1433Z	YWHAZ	1.46	−0.36	40/24	9194 (11,162)	12,024 (11,423)	0.76

Nineteen proteins had a VIPpred >1.0 and a p(corr) >0.35 and were able to discriminate between FM and CON at baseline. Six of 19 proteins were positively associated with FM (marked with *). Proteins in bold have more than one known isoform and were therefore grouped. For complete list of protein names, see Table S2 (Supplemental Digital Content, Appendix, http://links.lww.com/MSS/C433).

#### Protein pathway analysis for group differences at baseline

The 19 proteins from the OPLS-DA model were submitted to STRING pathway analysis, which resulted in a protein–protein interaction network that was significantly enriched (PPI enrichment, *P* < 0.01), containing 16 proteins (three immunoglobins were not identified in the STRING database). In the network, eight protein interactions were found, with an average local clustering coefficient of 0.46. Five enriched biological processes were identified with a FDR <0.05, which were response to stress, regulation of mRNA stability, and three processes related to immunity: defense response to gram-positive bacterium, opsonization, and complement activation (lectin pathway). Nine of the 16 proteins were included in the biological process of response to stress (Fig. 1C). The six proteins that were positively associated with FM were distributed among all processes.

#### Exercise-related proteomics (FM and CON)

**FIGURE 2:**
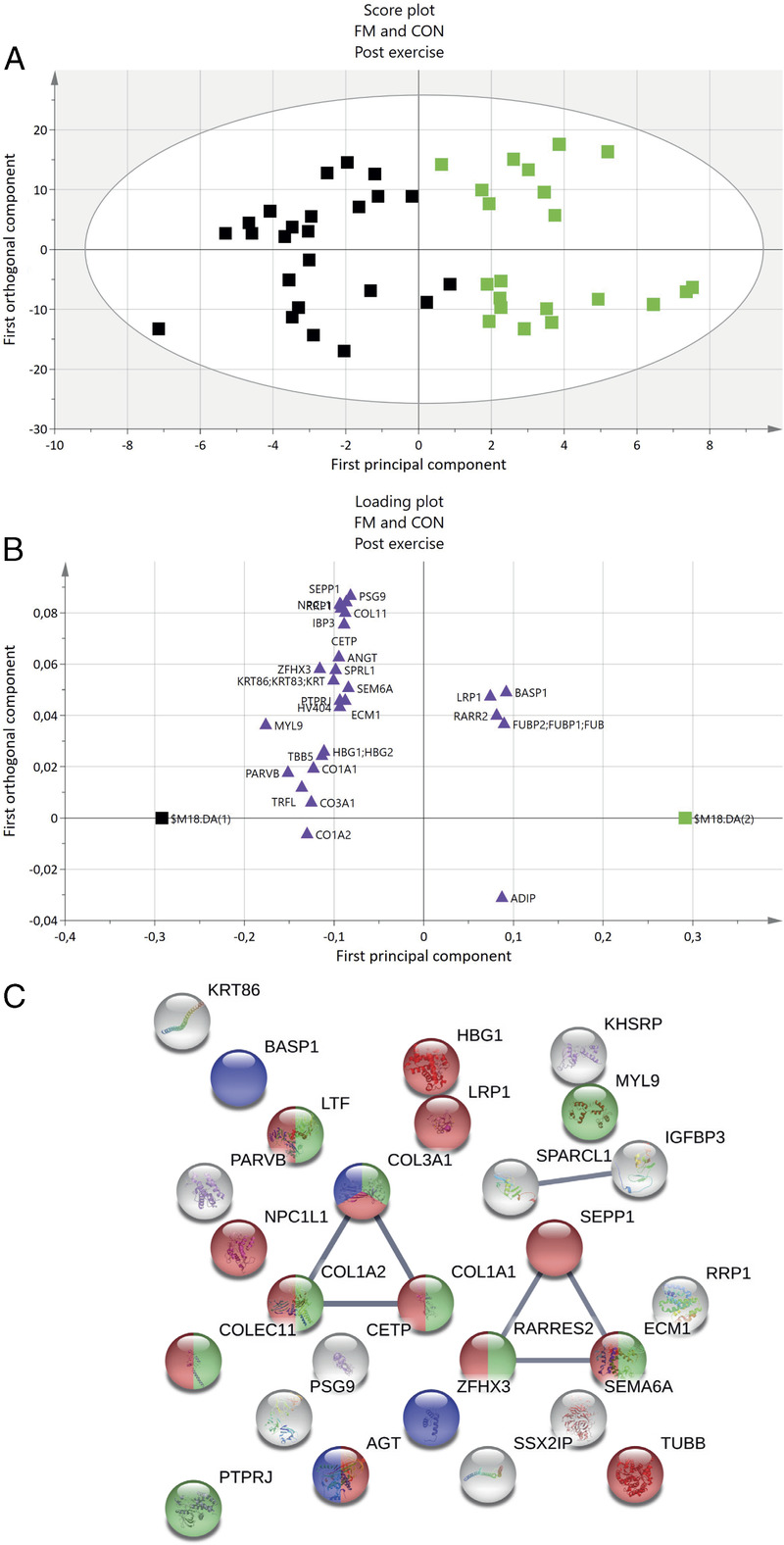
OPLS-DA model of FM and CON after exercise, and STRING protein network. A, The score plot visualizes the group separation between FM and CON: green, FM; black, CON. B, The loading plot shows significant protein with a VIPpred ≥1.0 and p(corr) ≥0.35 (purple triangles). In total, 28 proteins were significantly important for group separation. Five of 28 proteins had a positively association with FM. C, Enriched protein network of significant proteins. Node colors represent the following: green, regulation of immune system process; red, response to stress; blue, muscle structure development. Abbreviated protein names in loading plot and STRING network correspond to the same names in Table 4 and Table S2 (Supplemental Digital Content, Appendix, http://links.lww.com/MSS/C433).

To explore plasma protein changes after exercise compared with before exercise, an OPLS-DA including FM and CON participants only performing resistance exercise was performed. The OPLS-DA model postexercise had one PC, two OC, *R*^2^ = 0.748, *Q*^2^ = 0.452, and CV-ANOVA *P* = 0.0069. The significant OPLS-DA model had 28 proteins with a VIPpred ≥1.0 and p(corr) ≥0.35. These proteins were regarded as significantly altered proteins important for group separation between FM and CON after exercise. The proteins with the highest VIPpred (≥1.7) were all downregulated proteins of myosin regulatory light polypeptide 9 (MYL9), beta-parvin, lactotransferrin, and three forms of collagen alpha chain, ZFHX3, and tubulin beta chain (Table 4). Of the 28 proteins, 5 were positively associated with FM and 23 were positively associated with CON (Figs. 2A, B).

**TABLE 4:** Significant proteins group differences between FM and CON after exercise.

Protein Name	Accession Number (Uniprot)	Protein Name in MVDA Models	Gene Name (STRING Analysis)	VIPpred	p (corr)	No. FM/CON Protein Is Detected	FM, Median (IQR) Intensity	CON, Median (IQR) Intensity	Fold Change FM/CON
Myosin regulatory light polypeptide 9	P24844	MYL9	MYL9	2.65	−0.70	11/11	3080 (4089)	9809 (5840)	0.31
**Beta-parvin**	**Q9HBI1**	PARVB	PARVB	2.28	−0.71	5/8	1462 (624)	2994 (3369)	0.49
**Lactotransferrin**	**P02788**	TRFL	LTF	2.05	−0.54	21/24	2,226,465 (2,766,798)	5,802,484 (7,070,728)	0.38
Collagen alpha-2(I) chain	P08123	CO1A2	COL1A2	1.96	−0.52	15/22	4327 (4729)	9184 (9086)	0.47
Collagen alpha-1(III) chain	P02461	CO3A1	COL3A1	1.88	−0.51	16/20	3886 (3888)	6793 (4161)	0.57
Collagen alpha-1(I) chain	P02452	CO1A1	COL1A1	1.85	−0.49	21/24	5732 (5235)	10,209 (11,923)	0.56
Zinc finger homeobox protein 3	Q15911	ZFHX3	ZFHX3	1.74	−0.46	21/24	450,572 (110,794)	563,444 (141,784)	0.80
Tubulin beta chain	P07437	TBB5	TUBB	1.70	−0.43	13/17	5712 (7492)	11,064 (35,939)	0.52
**Hemoglobin subunit gamma 1**	**P69891**	HBG1	HBG1	1.68	−0.60	7/10	7182 (4519)	13,770 (16,933)	0.52
**Keratin, type II cuticular Hb6**	**O43790**	KRT86	KRT86	1.52	−0.40	21/24	318,460 (219,886)	374,304 (200,661)	0.85
SPARC-like protein 1	Q14515	SPRL1	SPARCL1	1.47	−0.41	21/23	2762 (2244)	3922 (2125)	0.70
Angiotensinogen	P01019	ANGT	AGT	1.42	−0.38	21/24	973,363 (703,114)	1,183,479 (797,982)	0.82
**NPC1-like intracellular cholesterol transporter 1**	**Q9UHC9**	NPCL1	NPC1L1	1.41	−0.39	21/24	244,069 (144,275)	301,279 (91,207)	0.81
Immunoglobulin heavy variable 4–4	A0A075B6R2	HV404	—	1.41	−0.37	18/19	7425 (11,417)	11,246 (7860)	0.66
**Receptor-type tyrosine-protein phosphatase eta**	**Q12913**	PTPRJ	PTPRJ	1.41	−0.40	21/23	9482 (5782)	13,890 (26,066)	0.68
Ribosomal RNA processing protein 1 homolog A	P56182	RRP1	RRP1	1.40	−0.39	21/24	53,668 (29,686)	68,752 (22,793)	0.78
**Insulin-like growth factor-binding protein 3**	**P17936**	IBP3	IGFBP3	1.38	−0.38	21/24	137,168 (142,682)	218,604 (87,440)	0.63
*Brain acid soluble protein 1	P80723	BASP1	BASP1	1.38	0.59	8/10	5166 (1550)	2954 (3701)	1.75
*Far upstream element-binding protein 2	**Q92945**	FUBP2	KHSRP	1.35	0.35	21/24	45,347 (25,432)	36,827 (22,718)	1.23
Cholesteryl ester transfer protein	P11597	CETP	CETP	1.34	−0.37	21/24	20,470 (29,078)	34,813 (15,350)	0.59
Extracellular matrix protein 1	Q16610	ECM1	ECM1	1.32	−0.38	21/24	183,344 (160,208)	291,711 (222,074)	0.63
*Afadin- and alpha-actinin-binding protein	**Q9Y2D8**	ADIP	SSX2IP	1.32	0.37	21/24	562,200 (233,715)	446,229 (119,601)	1.26
**Collectin-11**	**Q9BWP8**	COL11	COLEC11	1.32	−0.36	21/24	10,304 (7093)	15,153 (6983)	0.68
Selenoprotein P	P49908	SEPP1	SEPP1	1.30	−0.36	21/24	131,732 (104,877)	192,129 (116,913)	0.69
**Semaphorin-6A**	**Q9H2E6**	SEM6A	SEMA6A	1.27	−0.35	21/24	454,799 (328,061)	655,015 (387,455)	0.69
**Pregnancy-specific beta-1-glycoprotein 9**	**Q00887**	PSG9	PSG9	1.23	−0.35	21/24	176,423 (264,773)	263,594 (160,575)	0.67
*Retinoic acid receptor responder protein 2	Q99969	RARR2	RARRES2	1.23	0.35	14/21	6397 (1587)	4934 (1975)	1.30
*Prolow-density lipoprotein receptor-related protein 1	Q07954	LRP1	LRP1	1.12	0.42	11/18	3463 (1474)	2689 (1414)	1.29

Twenty-eight proteins had a VIPpred >1.0 and a p(corr) >0.35, which were able to discriminate between FM and CON after exercise. Five of 28 proteins were positively associated with FM (marked with *). Proteins in bold have more than one known isoform and were therefore grouped. For complete list of protein names, see Table S2 (Supplemental Digital Content, Appendix, http://links.lww.com/MSS/C433).

#### Protein pathway analysis for group differences after exercise

The 28 proteins from the significant OPLS-DA model postexercise were submitted to STRING pathway analysis, which resulted in a protein–protein interaction network that was significantly enriched (PPI enrichment, *P* < 0.01), containing 27 proteins (immunoglobulin heavy variable 4–4 (HV364) was not identified by the STRING database). In the network, seven protein interactions were found, with an average local clustering coefficient of 0.30. Several enriched biological processes were identified with an FDR <0.05, such as regulation of immune system process, muscle structure development, and response to stress (Fig. 2C). The five proteins positively associated with FM were included in stress or muscle structure development processes.

#### Relative protein changes related to exercise in FM and CON

**FIGURE 3:**
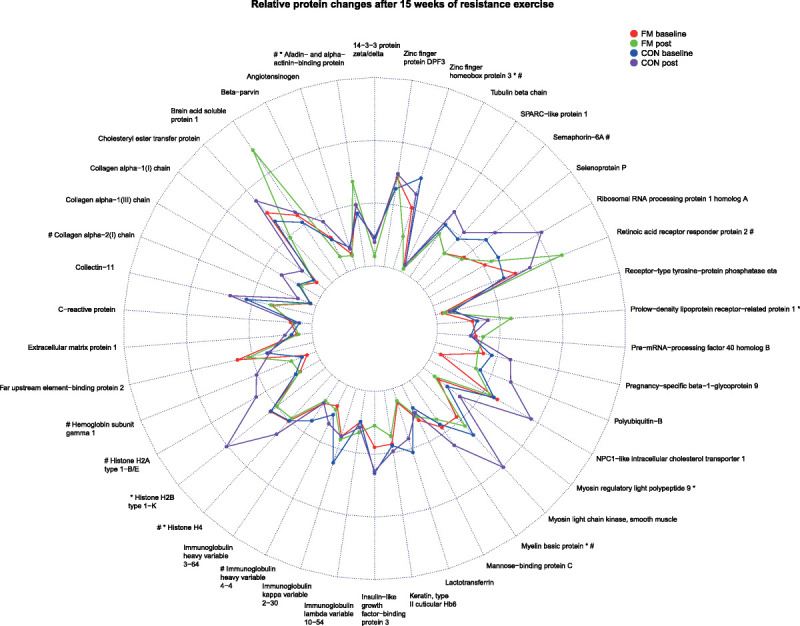
Changes of plasma proteins in FM and CON at baseline and after 15 wk of resistance exercise. Radar plot showing median relative protein intensities of significant proteins at baseline and postexercise for FM and CON. *P < 0.05 in FM group baseline vs post. #*P* < 0.05 in CON group baseline vs post.

**FIGURE 4:**
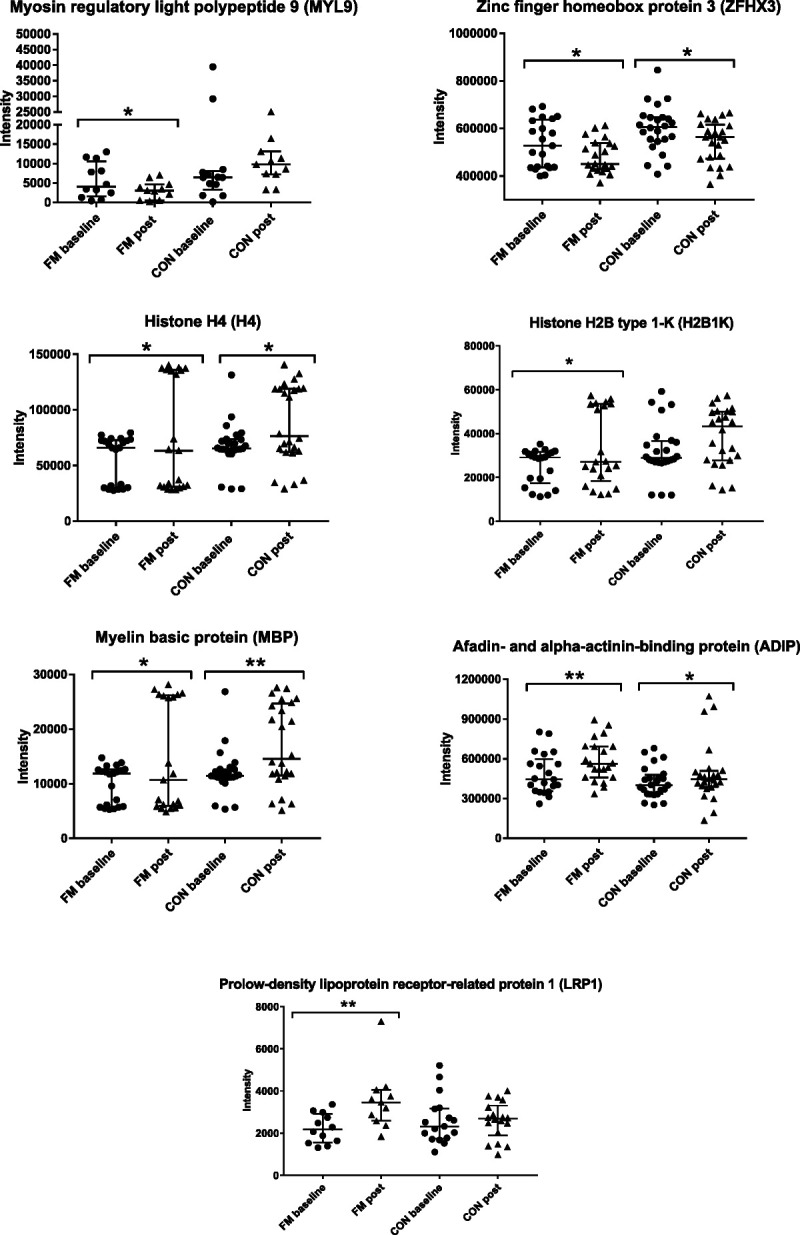
Significant changes in proteins after exercise in FM. Seven proteins were significantly altered in the FM group after 15 wk of resistance exercise when comparing protein intensity at baseline and postexercise. Out of these seven proteins, four was also significantly changed in the CON group. *P < 0.05; **P < 0.01.

To get an overview of the trends of direction of proteins and to analyze whether exercise had any effect on plasma protein changes in FM and CON, the significant proteins from both baseline and postexercise found in the two OPLS-DA models were analyzed with paired univariate statistics. Median relative protein changes for the two groups and time points are displayed in Figure 3. Out of the 47 proteins that were able to discriminate FM from CON (19 at baseline and 28 proteins at post), 7 proteins in FM and 10 proteins in CON were significantly altered within each group after 15 wk of resistance exercise. In FM, MYL9 (*P* = 0.049), ZFHX3 (*P* = 0.019), H4 (*P* = 0.027), histone H2B type 1-K (H2B1K; *P* = 0.023), and myelin basic protein (MBP; *P* = 0.027) were significantly reduced, and afadin- and alpha-actinin-binding protein (ADIP, *P* = 0.003) and prolow-density lipoprotein receptor-related protein 1 (LRP1; *P* = 0.005) were significantly increased after exercise (Fig. 4). In CON, four out of these seven proteins were significantly changed after exercise: ZFHX3 was reduced (*P* = 0.022), and MBP (*P* = 0.003), ADIP (*P* = 0.049), and H4 (*P* = 0.016) were upregulated. Other proteins that were significantly changed in CON were upregulated levels of Semaphorin-6A (SEM6A; *P* = 0.027), H2A1B (*P* = 0.011), hemoglobin subunit gamma 1 (HBG1; *P* = 0.027), HV364 (*P* = 0.035), collagen alpha-2(I) chain (CO1A2; *P* = 0.042), and retinoic acid receptor responder protein 2 (RARR2, *P* = 0.047).

#### Correlation of clinical characteristics and protein changes in FM after exercise

A significant OPLS model was obtained when regressing PPT and the significant proteins from postexercise (Table 4 and Fig. S1, Supplemental Digital Content, Appendix, http://links.lww.com/MSS/C433) in FM (model statistics: 1 PC, *R*^2^ = 0.44, *Q*^2^ = 0.31, CV-ANOVA *P* = 0.035). Forty proteins had a VIP ≥1.0 and p(corr) ≥0.75 and were associated with very low PPT, all negatively correlated with PPT in FM (Figs. S1a, b; Supplemental Digital Content, Appendix, http://links.lww.com/MSS/C433). The proteins with highest VIP (>1.27) and p(corr) (≥0.95) were NPC1-like intracellular cholesterol transporter 1, vitamin D–binding protein, pregnancy-specific beta-1-glycoprotein 9, gelsolin, insulin-like growth factor–binding protein 3, and MBP (Figs. S1a, b; Supplemental Digital Content, Appendix, http://links.lww.com/MSS/C433).

All 40 proteins were subjected to STRING analysis, resulting in a network including 39 proteins (killer cell immunoglobulin-like receptor 2DS5 was not identified by the STRING database). The analysis revealed a significantly enriched network (PPI enrichment, *P* = 9.04e−07) with a local average clustering coefficient of 0.43. Although several biological processes were identified (FDR <0.05), two processes covered almost all proteins: metabolic and immune system processes (Fig. S1c, Supplemental Digital Content, Appendix, http://links.lww.com/MSS/C433).

Other clinical variables were analyzed to investigate correlation with plasma proteins after exercise and pain intensity or HADS in FM; however, no significant OPLS models were obtained (data not shown).

#### Potential subgroups of FM related to clinical characteristics and exercise variables

A within-group separation could be seen in the FM group both at baseline (Fig. 1A, score plot) and postexercise (Fig. 2A, score plot). To get an overview and compare the proteome profile at baseline and postexercise in FM alone, a PCA was performed, including all proteins from both time points (Fig. S2, Supplemental Digital Content, Appendix, http://links.lww.com/MSS/C433). Based on the present two FM subgroups, analysis of background and exercise-related data showed a significant difference in FM duration, number of tender points, and mental fatigue at baseline. Postexercise, a significant difference in PPT was present between the two FM groups, and only subgroup 2 improved their physical strength in the majority of the exercise-related variables (Supplemental Digital Content file and Fig. S3, Supplemental Digital Content, Appendix, http://links.lww.com/MSS/C433).

#### OPLS modeling of FM subgroups and disease duration at baseline

To further evaluate the differences in the two FM subgroups, based on the differences in baseline characteristics, an OPLS model of FM duration and plasma proteins were analyzed to investigate whether any proteins correlated with FM duration. Two OPLS models were performed, one including subgroup 1 with shorter FM duration (median (IQR), 8 (7.8) yr) and one model including subgroup 2 with longer FM duration (median (IQR), 15 (6) yr; Supplemental Digital Content file and Fig. S4a, b; Supplemental Digital Content, Appendix, http://links.lww.com/MSS/C433). The significant proteins associated with FM duration were different between the two subgroups. Several of the significant proteins were involved in similar processes of stress. Hence, in subgroup 1 (shorter FM duration), the proteins were further involved in blood coagulation and immune response, whereas in subgroup 2 (longer FM duration), the proteins were involved in leucocyte-mediated immunity, immune system process, and inflammatory response (Fig. S4c, Supplemental Digital Content, Appendix, http://links.lww.com/MSS/C433). No significant models were obtained when regressing knee extension force or handgrip force variables for all FM or when divided into subgroups at baseline.

#### OPLS modeling of FM subgroups knee extension force after exercise

Because subgroup 2 had longer FM duration and showed improved physical capacity and muscle strength after exercise, two OPLS models investigating knee extension force (right and left sides) were performed. Several of the proteins that were associated with knee extension force in FM belonged to processes of wound healing, tissue development, and inflammatory response (Supplemental Digital Content file and Figs. S5a–c, Supplemental Digital Content, Appendix, http://links.lww.com/MSS/C433).

## DISCUSSION

This plasma proteomic study produced four major results:

- At baseline, 19 proteins involved in response to stress, regulation of mRNA stability, and immunity were able to discriminate between FM and CON.
- A potential subgroup division within the FM group was detected. Several plasma proteins were correlated with FM duration at baseline and knee extension force after exercise.
- After 15 wk of resistant exercise, 28 proteins involved in regulation of immune system process, muscle structure development, and response to stress were able to discriminate between FM and CON.
- Postexercise in FM, PPT significantly correlated with several proteins involved in metabolic and immune response processes.

To date, few studies have focused on plasma proteomics in FM. In this study, we first investigated whether any plasma proteins could be used to distinguish the FM group from healthy controls before a 15-wk exercise intervention. This was done in order to try to validate our previous findings on the same cohort analyzing the plasma proteome of FM using two-dimensional gel electrophoresis (2-DE) (36). The present investigation revealed 19 proteins involved in a variety of processes such as response to stress, regulation of mRNA stability, and different forms of immunity processes. Ramirez-Tejero et al. (33), in a study of the plasma proteome profile of FM and healthy controls, found several enriched biological processes possibly related to inflammation, specifically blood coagulation, acute-phase response, complement system, liver-X receptor/retinoid-X receptor activation, and farnesoid-X receptor/retinoid-X receptor activation in FM. Similar findings were found in a recent serum proteomic study by Han et al. (32), suggesting inflammation and blood coagulation processes are involved in the pathophysiology of FM. In our previous plasma proteomic study of FM using 2-DE, we found clear differences between FM and controls in proteins related to inflammation, metabolic, and immunity processes (36). In another study, we found that proteins involved in the complement system, and metabolic and inflammatory processes were altered in CWP/FM patients compared with controls (34). Ruggiero et al. (35) examined the serum proteome of FM and controls, and found alterations of the proteins transthyretin, alpha-1-antitrypsin, and retinol-binding protein 4. However, in this study, we were unable to confirm the exact same discriminating proteins as described in the aforementioned studies. Nonetheless, several other proteins were detected and related to similar processes as previously seen, such as immunity processes and complement system activation (Table 3). Among the processes seen in the plasma/serum proteomic studies of FM (32–34,36), immunity processes and complement activation were also found enriched in the pathway analysis performed in the systematic review by Gerdle and Ghafouri (46). Overall, these studies, together with the results from this study, point toward an alteration of different plasma proteins in FM, especially proteins involved in the immune response.

In addition, a clear subgroup division among the FM group was noted in the MVDA models. Additional analysis of the background data between these two groups at baseline and postexercise revealed that several clinical variables were different between the two subgroups and that there might be different protein processes activated systemically, pointing toward FM patients with longer FM duration (subgroup 2) who seem to have a more immunity/inflammatory profile at baseline (Figs. S2–S4, Supplemental Digital Content, Appendix, http://links.lww.com/MSS/C433). One reason for the improvement in muscle function seen in subgroup 2 could be attributed to better preconditions (e.g., a lower number of tender points were seen in subgroup 2). A reduction in tender points might reflect that this subgroup is more susceptible to perform exercise because they do not have as many painful sites that could hinder them from performing exercise intervention. At baseline, both subgroups had lower strength in their muscles, which could reflect a general low exercise level to start with and a lower muscle function. This lower strength could be one reason for the improvement seen in the muscle strength variables in FM at a group level.

This study shows that exercise has beneficial effects on FM patients as several of the clinical and exercise-related variables improved (Table 2). These results and other parameters have been reported elsewhere; hence, not the exact same number of participants was reported (11,12,20). Other studies have found improvement of clinical variables in FM after different forms of exercise regarding pain intensity, fatigue, quality of life, psychological distress, muscle strength, and physical functioning (9,47).

Although exercise is considered as one of the major treatment options for FM, few studies have explored protein biomarkers related to exercise in FM. To the best of our knowledge, this is the first proteomic study exploring changes in plasma proteins in FM and CON after 15 wk of resistance exercise. Overall, several proteins seem to be affected by exercise in both FM and CON. Out of the 47 significant proteins in the OPLS models (5 protein were shared among both models: COLL11, FUBP2, NPCL1, RRP1, ZFHX3), only 7 proteins in FM reached statistically significant changes after exercise (ADIP, LRP1, ZFHX3, H2B1K, H4, MBP, MYL9; Fig. 4). Even though few proteins seem to be affected by exercise in the FM group (the more in CON), the trends of changes in the proteins moved in the direction of baseline levels of controls and toward a normalization (Fig. 3), for example, extracellular matrix protein 1, several forms of collagens, polyubiquitin-B, Far upstream element-binding protein 2, histones, different forms of immunoglobulins, mannose-binding protein C, and myosin light-chain kinase (smooth muscle). Among the proteins positively associated with FM at baseline (CRP, DPF3, PR40B, FUBP2, MBL2, LVX54) and postexercise (BASP1, FUBP2, ADIP, RARR2, LRP1), LRP1 was one of the proteins that was significantly increased in FM and exceeded the levels of CON at both preexercise and postexercise. LRP1 is a multifacet protein receptor involved in lipoprotein endocytosis, intracellular signaling, and phagocytosis, and may be involved in regulation of several inflammatory pathways (48). In neuropathic pain, activation of LPR1 has shown to be able to reduce inflammatory response by reducing the production of proinflammatory mediators through different signaling pathways (49). The involvement of LRP1 in FM is unknown, but it is tempting to speculate that the increase in this protein after exercise has a protective role in FM, as it potentially plays a role in modulating and regulating inflammatory response.

Previous proteomic studies in healthy subject have found that exercise is related to alterations in proteins involved in processes of immune response, inflammation, cellular stress signaling, coagulation, glycolytic and metabolic enzymes, and contractile and structural proteins (29,30,50,51). Although these studies are difficult to compare with our study because of their small cohorts, health status, sex of subjects, and type of exercise performed, it is not surprising that similar processes are identified in our study (stress response, muscle structure development, and immunity processes). Further investigations are needed to evaluate whether the present protein changes after exercise are related to the intervention or to the disease itself.

In three proteins (H4, H2B1K, MBP), a cluster of eight FM patients who were included in subgroup 1 were seen (shorter FM duration and no improvement in exercise-related variables). Although at a group level these proteins were downregulated after exercise, these eight FM patients showed the opposite. The increase of these proteins in this subset of FM after exercise might be related to shorter FM duration or lower PPT. Hence, MBP was one of the proteins with the highest VIP value and associated with lower PPT in FM after exercise. The effect and function of these proteins related to specific subsets of FM and exercise need further investigations.

There was a significant difference in PPT between the two FM subgroups after exercise, although at a group level no improvement in pain sensitivity existed. Lower PPT in FM was found to be associated with proteins involved in immunity, inflammation, and blood coagulation processes in our previous study (36). Plasma proteins related to immune response and transport were also found associated with PPT in CWP/FM (52), and from the same cohort, the trapezius muscle proteome revealed proteins involved in stress, and metabolic and inflammatory processes to be correlated with PPT in CWP/FM (53). In this study, we confirmed that the same processes (i.e., metabolic and immune responses) were associated with PPT after exercise. Hence, overall the FM group improved their muscle strength in some variables, but their pain sensitivity remained, reflecting that exercise did not affect the pain sensitivity or proteins related to PPT in FM. The fact that we confirmed the same processes in two cohorts further adds to the finding that specific protein patterns are associated with PPT in FM.

Furthermore, the knee extension force variable was additionally correlated with plasma proteins involved in wound healing, inflammatory response, and tissue development after exercise (Fig. S5c, Supplemental Digital Content, Appendix, http://links.lww.com/MSS/C433). The proteins correlated with improved muscle force in the quadriceps muscle and the enriched processes might reflect trends of changes in the muscles that are reflected systemically after exercise in FM. Further studies that examine the implications of this finding and why this specific FM subset benefits more than other FM subsets from resistance exercise therapy are needed.

Several of the previously published studies on the same cohort have investigated selected cytokines (20,21), metabolites (26), neuropeptides (54), and endocannabinoids (27). The effect of resistance exercise on these circulating molecules have been sparse; only IL-1ra, glutamate, pyruvate, glucose, anandamide, and stearoylethanolamide were changed after exercise, findings that suggest a change in the metabolic and inflammatory lipid profile in FM after exercise. Taken together, the significant proteins and associated enriched protein networks seen at baseline and postexercise in FM in this study suggest there are several ongoing mechanisms involved and that these processes, to some extent, could be modulated by exercise in FM.

Several limitations exist in this study. There was a significant difference in BMI between FM and CON, which could have influenced the results. However, this difference was not present between FM and CON in participants only performing resistance exercise but could have influenced the results at baseline reflecting pain-related proteomics. In addition, several of the patients in the FM group were on regular medication, which also could have influenced the proteomic results. Because we used LC-MS/MS–based proteomics, not 2-DE, the study of proteoforms was difficult, which we have shown to be important in our previous proteomic studies of CWP/FM (34,36). In future longitudinal proteomic studies of FM and interventions, machine learning techniques should be applied to evaluate potential biomarkers that are disease and intervention specific. Hence, the analytical approach of this study is more explorative because, to the best of our knowledge, there are no other studies that have investigated protein changes in FM in relation to resistance exercise previously.

## CONCLUSIONS

In conclusion, because of the complex nature of FM with the presence of a variety of symptoms and unknown etiology, it is highly unlikely that only one biomarker could explain the whole pain condition or could be used for diagnostic proposes. Therefore, the study of the plasma proteome has great potential to evaluate potential underlying mechanisms and biomarkers and to evaluate different interventions such as resistance exercise used for FM. This study has shown prominent differences in the plasma proteome profile between FM and CON at baseline. Fifteen weeks of resistance exercise improved several of the clinical and exercise-related variables, and for the first time, the plasma proteome has been investigated in FM after 15 wk of resistance exercise. Several protein changes existed related to immunity, stress, mRNA stability, muscle structure development, and metabolic processes both at baseline and postexercise. Hence, exercise seems to affect circulating proteins, clinical characteristics, and muscle strength in FM. Finally, it seems that there are specific plasma proteome profiles related to PPT, and specific subgroups of FM based on FM duration and improved muscle force in the quadriceps muscle. This study further contributes to better understanding of systemic protein changes in women with FM and the effects of resistance exercise on such changes.
